# Distinctive pattern mapping between cardiometabolic multimorbidity and neuropsychiatric disturbance in multi‐regional‐ethnical older adults

**DOI:** 10.1002/alz.085316

**Published:** 2025-01-09

**Authors:** Haoran Zhang, Haoxuan Wen, Xuhao Zhao, Christopher Chen, Shifu Xiao, Zuyun Liu, Xin Xu

**Affiliations:** ^1^ School of Public Health and the Second Affiliated Hospital of School of Medicine, Zhejiang University, Hangzhou, Zhejiang China; ^2^ The Key Laboratory of Intelligent Preventive Medicine of Zhejiang Province, Hangzhou, Zhejiang China; ^3^ School of Public Health, the Second Affiliated Hospital of School of Medicine, Zhejiang University, Hangzhou, Zhejiang China; ^4^ Memory Aging and Cognition Center, National University Health System, Singapore Singapore; ^5^ Yong Loo Lin School of Medicine, National University of Singapore, Singapore Singapore; ^6^ Shanghai Mental Health Center, Shanghai Jiao Tong University School of Medicine, Shanghai China; ^7^ Memory, Ageing, and Cognition Centre (MACC), Department of Pharmacology, Yong Loo Lin School of Medicine, National University of Singapore, Singapore, Singapore Singapore

## Abstract

**Background:**

Presence of cardiometabolic multimorbidity (CMM) has been linked to depressive symptoms in adults. The present study aimed to investigate the distinctive mapping between CMM and differential neuropsychiatric subsyndromes among multi‐regional and ethnical older adults.

**Method:**

The present study included discovery and validation datasets. The discovery dataset consisted of two longitudinal and two cross‐sectional studies from Asian older adults from both clinical and community settings. The longitudinal validation dataset was from the UK Biobank (UKB). CMM was defined as the coexistence of ≥2 conditions including hypertension, hyperlipidaemia, diabetes mellitus, stroke, and other cardiovascular diseases (CVD). Subsequently, two CMM patterns, including metabolic (≥2 of hypertension, hyperlipidaemia and diabetes mellitus) and cardio‐cerebrovascular multimorbidity (≥2 of hypertension, stroke and CVD) were identified respectively. The neuropsychiatric inventory (NPI) was applied to define four neuropsychiatric subsyndromes in the discovery dataset: psychosis, hyperactivity, affective and apathy. In the validation dataset, the four subsyndromes were defined using previously established methods. Short‐term incidence of subsyndromes was defined as the development of neuropsychiatric symptoms (NPS) in recent 1‐2 years. Two‐step individual participant data was used to establish the cross‐sectional association between CMM and NPS. Cox regression models explored baseline CMM patterns and NPS incidence in both discovery and validation datasets.

**Result:**

The discovery dataset included 2950 Asian participants for cross‐sectional analysis and 967 participants (675 NPS‐free at baseline) for longitudinal analysis. The validation dataset included 885 participants (765 NPS‐free at baseline) for longitudinal analysis. People with CMM had a higher presence of NPS and a higher risk of 1‐year incidence of affective syndrome (hazard ratio [HR] = 3.95, 95%CI = 1.44‐10.82). Regarding CMM patterns, metabolic multimorbidity was associated with affective syndrome at baseline (odds ratio [OR] = 1.35, 95%CI = 1.01‐1.81), as well as its short‐term incidence (HR = 3.88, 95%CI = 1.53‐9.85). In the validation dataset, CMM, especially metabolic multimorbidity, was also associated with an increased risk of short‐term incidence of affective subsyndrome (HR = 8.04, 95%CI = 2.65‐24.40, and HR = 6.44, 95%CI = 2.11‐19.62, respectively). Cardio‐cerebrovascular multimorbidity was not associated with incidence of any NPS in both datasets.

**Conclusion:**

CMM, especially metabolic multimorbidity was associated with affective disturbances in multi‐regional‐ethnical older adults. Our findings emphasized the importance of targeted management of metabolic multimorbidity.

